# Improvement of *Undaria pinnatifida* Sugar-Free Gummy Jellies’ Properties by Phycocyanin Under Ultraviolet (UV) Irradiation

**DOI:** 10.3390/foods13243988

**Published:** 2024-12-10

**Authors:** Ying Bai, Yihan Sun, Chenglei Qiu, Wenxin Xiang, Yu Liu, Yujiao Wang, Hang Qi

**Affiliations:** National Engineering Research Center for Seafood, State Key Laboratory of Marine Food Processing and Safety Control, Collaborative Innovation Center of Provincial and Ministerial Co-Construction for Seafood Deep Processing, Liaoning Province Collaborative Innovation Center for Marine Food Deep Processing, Dalian Technology Innovation Center for Chinese Pre-Made Food, College of Food Science and Technology, Dalian Polytechnic University, Dalian 116034, China; baiying0212@163.com (Y.B.); 18641423774@163.com (Y.S.); qcl163163@163.com (C.Q.); 13624089758@163.com (W.X.); liuyu01622@163.com (Y.L.); wangyj202012@163.com (Y.W.)

**Keywords:** *Undaria pinnatifida*, phycocyanin, gummy jelly, texture characteristics, accelerated storage

## Abstract

In this study, *Undaria pinnatifida* (UP) was used as the primary research material, and sugar-free gummy jelly was prepared using ultraviolet (UV) irradiation with phycocyanin. The properties were measured using a texture analyzer, color difference analyzer, low-field nuclear magnetic resonance (LF-NMR) analyzer, and sensory evaluation. Additionally, the stability during accelerated storage was examined. The results showed that UV irradiation-assisted phycocyanin significantly increased the hardness of the sugar-free gummy jelly, from 268.4 ± 11.0 g to 477.9 ± 5.2 g, and enhanced its chewiness, from 247.4 ± 12.2 to 415.1 ± 3.1. Additionally, the jelly exhibited stronger water binding ability, with the proportion of immovable water increasing from 6.17 ± 0.66% to 9.52 ± 0.77%. During accelerated storage, the texture properties, color, water migration, and phycocyanin content of the sugar-free gummy jelly were changed. However, UV irradiation-assisted phycocyanin treatment slowed down the changes in the texture, color, and phycocyanin content of the sugar-free gummy jelly, which indicated that the product had good stability during storage. These results enhance the application of UP in sugar-free gummy jellies.

## 1. Introduction

In recent years, there has been a growing demand for healthier food alternatives, particularly in the confectionery industry. Sugar-free products are gaining popularity due to the increasing awareness of the health risks associated with excessive sugar consumption, such as obesity, diabetes, and cardiovascular diseases. Gummy jellies, a popular snack among both children and adults, can be created in a range of flavors and colors [1] but are typically high in their sugar contents. Currently, most sugar-free gummy jellies on the market use substitutes like xylitol, erythritol, and others to replace white sugar, aiming to meet the consumer demand for healthier diets. However, true zero-added-sweetener gummy jellies are rare. The main technical challenge lies in the fact that when only a gelling agent is used, the resulting texture is too soft, and the taste is subpar, making it difficult to meet consumer expectations for the texture and flavor of gummy candies. Therefore, developing sugar-free alternatives that retain desirable sensory properties such as texture and taste has become a key focus of food researchers and manufacturers. 

*Undaria pinnatifida* (UP), a form of marine brown algae, has continuously been a part of the food consumed by people, notably those in Asian countries such as China, Japan, and South Korea [2], and thrives well in warm gulfs. It is both highly delicious and medicinal [3]. In addition, it is rich in a variety of nutrients, including dietary fiber, minerals, vitamins, and polysaccharides. It possesses multiple health benefits, such as antioxidant, anti-inflammatory, anti-tumor, and immune-regulating properties. Additionally, it can help reduce blood lipids and blood sugar, can promote intestinal health, and is widely used in food processing. UP is commonly used in soups, salads, sushi, and seafood dishes and is often an essential ingredient in functional foods. However, its application in sugar-free gummy jelly formulations remains underexplored. When only UP pulp is used as the raw material to make gummy jellies, the resulting products tend to have a soft texture, lacking in elasticity and chewiness, due to the weak gelling properties of the pulp. Additionally, during storage, the gummy jellies are prone to significant water migration and loss, leading to dryness, hardening, or a loss of elasticity. Environmental factors such as temperature and humidity fluctuations can also cause changes in the texture and color, which can negatively affect market acceptance. Although UP pulp is rich in nutrients, its application in gummy jellies may not significantly enhance the overall nutritional value of the product, especially in the absence of other functional ingredients, with limited nutritional gains.

Ingredients like fiber, antioxidants, vitamins, and hypoglycemic sweeteners are easy to put into gummy jelly products to boost their nutritional content without losing their attraction to customers [4,5,6]. Previous studies examined the enrichment of candies with diverse food-derived bioactive components such as betaxanthins [7] and diverse plant-based extracts including *Psidium guajava* [8], mountain germander [9], beetroot pomace [10], *Cudrania tricuspidate* [11], *Clitoria ternatea flower* [12], red pitaya fruit puree [13], and red beet [5]. Phycocyanin, a natural blue pigment that absorbs light energy [14] and is present in *Spirulina platensis*, has been identified for its antioxidant, anti-inflammatory, anti-cancer, and gut-protective qualities [15]. It is widely used as a natural colorant in food products and is considered a promising bioactive compound for enhancing the nutritional profile of food products.

UV irradiation is a low-energy, low-cost food processing method. It is frequently employed in food safety control because it has the ability to deactivate micro-organisms by destroying their nucleic acids [16]. At the same time, UV irradiation can produce singlet oxygen, superoxide free radicals, and hydroxyl free radicals; drive protein structure development; and promote crosslinking [17]. However, how UV irradiation interacts with phycocyanin and UP pulp to influence the quality of gummy jelly remains unclear.

Therefore, the aim of this study was to evaluate the effect of phycocyanin and UV irradiation-assisted phycocyanin on UP sugar-free gummy jellies’ properties. More specifically, sugar-free gummy jellies were prepared using UP pulp as the raw material, with UV irradiation-assisted phycocyanin, and evaluated through a sensory analysis, water distribution analysis, and bacterial count. In addition, the physicochemical changes to the sugar-free gummy jellies, including their texture, color, water distribution, and phycocyanin content, were determined in an accelerated storage process. Through this study, we hope to provide new ideas for the development of UP in gummy jellies and promote the application of UV irradiation-assisted phycocyanin in the food industry.

## 2. Materials and Methods

### 2.1. Materials and Reagents

Salted UP (about 3 kg) was acquired from Dalian Mariculture Group Co., Ltd. (Dalian, China), and kept at 4 °C. Phycocyanin (PC, E25, 99% purity) was purchased from Zhejiang Binmei Biotechnology Co., Ltd. (Linhai, China). Potassium sorbate (food grade) was obtained from Henan Wanbang Industrial Co., Ltd. (Shangqiu, China); gelatin (Type B) was obtained from Beijing Baoxidi Technology Co., Ltd. (Beijing, China). No additional purification was required before using any other chemicals.

### 2.2. Preparation of UP Pulp

The salted UP was soaked for 15 min in deionized water before being washed three times to remove the salt. Unstained verdant green UP was selected for the preparation of the pulp. After that, deionized water was mixed with UP in a 2:1 (*w*/*v*) ratio before it was ground into UP pulp.

### 2.3. Preparation of Sugar-Free Gummy Jellies

**Figure 1:**
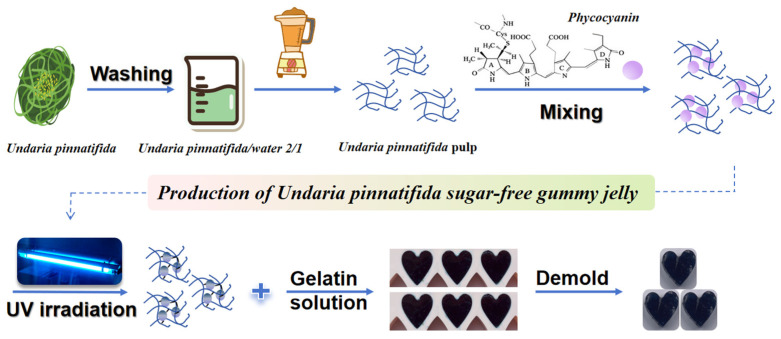
Production of *Undaria pinnatifida* sugar-free gummy jelly.

UP pulp and phycocyanin solution were mixed at a ratio of 10:1 (*w*/*w*) and treated with UV irradiation for 60 min [18]. Then, a certain amount of gelatin powder was added to a certain amount of deionized water and was stirred in a constant-temperature magnetic agitator at 60 °C for 30 min to ensure that the gelatin solution dissolved and the gelatin solution concentration was 20%. The gelatin solution and the above mixture were mixed hot at a ratio of 25:75 (*w*/*w*), then 0.05% potassium sorbate was added, and the mixture was immediately poured into a mold and cooled to room temperature for use. Three groups were set up: (1) without UV irradiation treatment and phycocyanin (control group); (2) without UV irradiation treatment and with phycocyanin (PC group); (3) with UV irradiation treatment and phycocyanin (UV-PC group). The production process of UP sugar-free gummy jelly is shown in Figure 1.

### 2.4. Sensory Evaluation

A sensory evaluation of the sugar-free gummy jellies was carried out in accordance with the approach described by [19]. Twenty participants (ten males and ten females) between the ages of 21 and 30 were chosen as professionally qualified evaluators. All of the testers were within a normal weight range (BMI < 30 kg/m^2^) and did not suffer from any allergies, diseases, or functional impairments that would impact their judgment or sensory perception. Food preferences, dental braces, diabetes, and pregnancy were also considered exclusion factors. There was no requirement for formal ethics authorization for this investigation. We made sure the internal participants in the experiment were informed about it, willing to participate, and safe before we began. The participants gave their informed consent for the collection of all data utilized in this study, and they were also informed of how the data would be used.

Color, texture, flavor, and appearance were the sensory evaluation parameters for the sugar-free gummy jelly. Each criterion was worth 25 points, and the total score for the sugar-free gummy jellies was based on a 100-point scale. The test was conducted at room temperature.

### 2.5. Low-Field Nuclear Magnetic Resonance (LF-NMR) Analysis

We examined the distribution of moisture and its interactions within the sugar-free gummy jellies using a low-field nuclear magnetic resonance (LF-NMR) analyzer (MesoQMR23-060H, Suzhou Niumai Electronic Technology Co., Ltd., Suzhou, China), with a particular emphasis on the critical role that moisture plays in determining the texture and quality of the sugar-free gummy jellies, following the approach of [20], with minor changes. T_2_ was determined by the Carr Purcell Meiboom Gill sequence, with key parameters set as follows: time echo (TE), 0.5 ms; time wait (TW), 4000 ms; number of scans (NS), 8; and number of echoes (NECH), 8000.

### 2.6. Determination of Total Bacterial Count (TBC) and Total Coliform Count (TC)

The sugar-free gummy jellies were stored at 4 °C for 7 days for TBC and TC determinations. The plate count method was used according to a previous publication [21].

### 2.7. Physicochemical Changes in the Accelerated Storage Process

Accelerated storage testing is frequently performed to study product stability under long-term storage at higher temperatures and relative humidity [22]. The storage stability of the sugar-free jellies was evaluated by measuring the texture, color, water state, and phycocyanin content during different storage days according to the method of [23], with minor changes. The experimental conditions were maintained at 37 °C and 60% humidity using an incubator with a consistent temperature and humidity. After 0, 4, 8, 12, and 16 days, samples were taken to evaluate their texture, color, water state, and phycocyanin content.

#### 2.7.1. Texture Profile Analysis (TPA)

The textural characteristics of the sugar-free gummy jellies were determined using a texture analyzer (TA-XT plus, Stable Micro Systems Ltd., Vienna, UK). A sample was put on the platform and the instrument was adjusted to the proper settings before measuring. The P/50 probe model was used to conduct TPA at room temperature. The pre-test speed was 2 mm/s, the test speed was 1 mm/s, the post-test speed was 2 mm/s, the strain was 20%, the compression period was 5 s, and the trigger force was 5 g. The hardness (N), gumminess, chewiness, springiness, cohesiveness, and resilience were calculated automatically by the test software (V4.0.6.0).

#### 2.7.2. Color Measurement

The sugar-free gummy jellies were cut into 5 mm samples and tested for brightness (L*), redness to greenness (a*), and yellowness to blueness (b*) using a chromameter (UltraScan Pro, HunterLab, Reston, VA, USA). The values of L*, a*, and b* were obtained. The color difference, ΔE, was calculated by Equation (1):

(1) $$∆E=\sqrt{{\left(∆L*\right)}^{2}+{\left(∆a*\right)}^{2}+{\left(∆b*\right)}^{2}}$$

#### 2.7.3. LF-NMR Analysis

The water state of the sugar-free gummy jellies during accelerated storage was measured according to the method of 2.5.

#### 2.7.4. Determination of the Phycocyanin Content

To quantify phycocyanin, 0.5 g of each sugar-free gummy jelly was crushed and dissolved in 5 mL water. After ultrasonic treatment at 25 °C for 20 min, the insoluble matter was removed by centrifugation at 8000 rpm for 5 min. The content of phycocyanin in the samples was detected using a microplate reader (Tecan 200, Hombrechtikon, Switzerland) according to a previous method [24]. The absorbance value at 620 nm was measured. The phycocyanin content was calculated by regression Equation (2):

(2) y=1.6374x+0.0609

where x is the phycocyanin mass concentration, mg/mL; y is the absorbance value of the sample solution at 620 nm.

### 2.8. Statistical Analysis

All tests were performed in triplicate, and the results are presented as the mean ± standard deviation (SD). A one-way analysis of variance was carried out on the data using SPSS 22 (SPSS Inc., Chicago, IL, USA) statistical analysis software. *p*-values of <0.05 were considered statistically significant.

## 3. Results and Discussion

### 3.1. Sensory Evaluation Analysis

**Figure 2:**
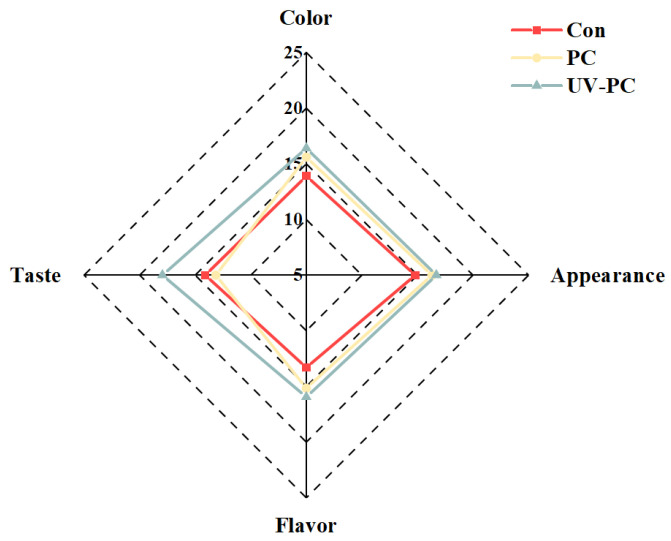
Sensory evaluation of *Undaria pinnatifida* sugar-free gummy jellies.

Sensory evaluation is a significant tool that must be taken into account when assessing the potential of ingredients to strengthen food formulations and the acceptance of the final product [25]. Food healthiness is the most important factor influencing customer desire, followed by flavor, taste, and color, according to a prior study [26]. The effects of phycocyanin and UV irradiation on the sensory properties of the sugar-free gummy jellies are shown in Figure 2. In terms of flavor, it was observed that with the addition of phycocyanin and UV irradiation treatment, the scores of the samples undoubtedly increased; this suggested that the different sample variations may be mainly attributable to sensory interactions between flavors. The flavors were perceived differently as a result of this interaction, which produced the observed variations [27]. Further, with regard to the color and appearance properties, the scores of the PC group and the UV-PC group were similar and were significantly higher than that of the control group, indicating that the addition of phycocyanin and UV irradiation treatment made the color more vivid and improved the appearance of the sugar-free gummy jellies. Lastly, in terms of taste, the sugar-free gummy jellies treated by UV irradiation had a higher taste score, indicating that they had good palatability. This was mainly because UV irradiation increased the density of the gel network structure in the sugar-free gummy jellies with phycocyanin, hardened their texture, reduced their viscosity, reduced their stickiness to the teeth, and improved their overall acceptability.

### 3.2. Water Migration of Sugar-Free Gummy Jellies After UV Irradiation

The transverse relaxation time (T_2_) and T_2_ peak proportion (P_2_) reflect the water distribution within a sample and are two significant outcome markers of water migration [28]. The T_2_ value represents the aqueous phase’s molecular mobility. The correlation between the degree of immobilization and the molecular mobility of the aqueous phase is inversely proportional. The T_2_ band can be further described as the three peaks representing three different water states. They are represented by bound water T_21_, immovable water T_22_, and free water T_23_ [29]. The proportions of these three peaks are evaluated by P_21_, P_22_, and P_23_, which describe how the content of each water component is distributed.

**Figure 3:**
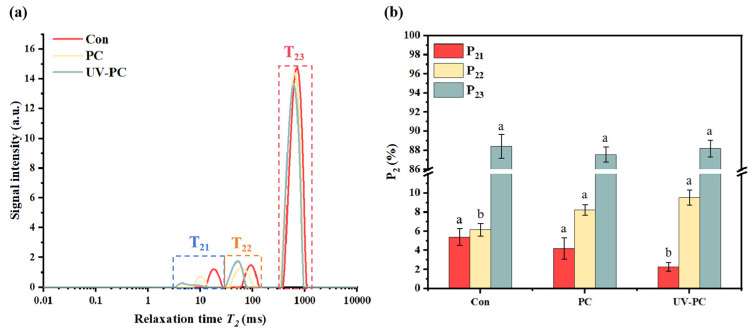
The water migration curves of *Undaria pinnatifida* sugar-free gummy jellies: (**a**) transverse relaxation time T_2_ curve; (**b**) peak ratio P_2_ diagram. Different lowercase letters indicated significant differences (*p* < 0.05).

As presented in Figure 3a, it is observed that the T_21_, T_22_, and T_23_ values of sugar-free gummy jellies in the UV-PC group were significantly lower compared to those in the control group and the PC group. This difference is most likely due to the hygroscopic nature of phycocyanin and the UV irradiation treatment, which led to the constant migration of water towards immobility and reduced the freedom of water [30]. Phycocyanin interacted with different structural characteristic components of the UP pulp under UV treatment, which additionally raised the relative number of polymers and eventually inhibited water movement [30]. Figure 3b shows the distribution of water in the three states. The predominant form of water within the sugar-free gummy jellies was free water. With the addition of phycocyanin and UV irradiation treatment, the content of free water changed but the results were not significant, while the content of immovable water in the samples increased significantly. This difference can likely be attributed to the gel network structure of the UV-PC group being denser and having an enhanced ability to bind water [31].

### 3.3. TBC and TC Evaluation

**Figure 4:**
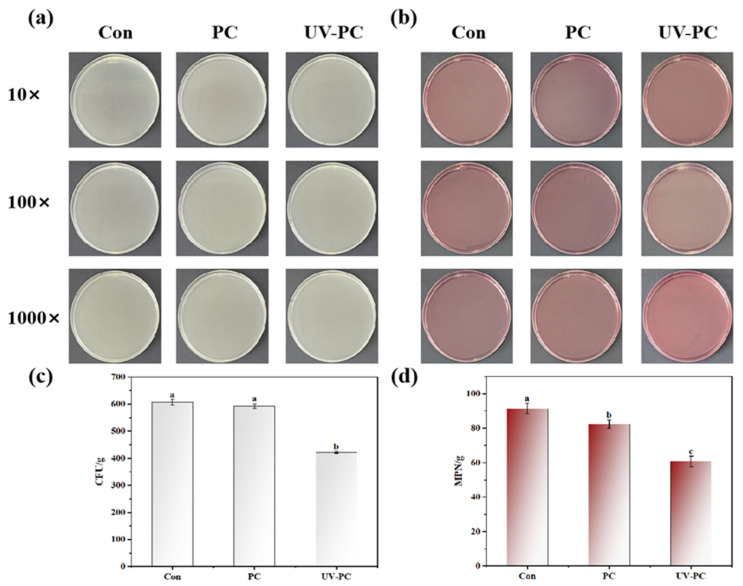
CFU and MPN of *Undaria pinnatifida* sugar-free gummy jellies: (**a**) appearance of CFU; (**b**) appearance of MPN; (**c**) quantitative analysis of CFU; (**d**) quantitative analysis of MPN. Different lowercase letters indicated significant differences (*p* < 0.05).

To guarantee the product’s safety, it is crucial to start with high-quality TBC and TC [21]. The TBC and TC of gummy jellies are limited to 10^5^ and 10^2^, respectively, according to the relevant national standards. As shown in Figure 4a,c, the TBCs of the three kinds of sugar-free gummy jellies were 607.3 ± 10.0 CFU/g, 592.7 ± 9.1 CFU/g, and 422.3 ± 3.1 CFU/g, respectively. After 7 days of storage, the TBCs remained within the detection limit, as expected. Such values confirmed the good quality of the sugar-free gummy jellies.

In Figure 4b,d, the TCs of the three kinds of sugar-free gummy jellies were 91.3 ± 3.1 MPN/g, 82.3 ± 2.5 MPN/g, and 60.7 ± 3.1 MPN/g, respectively. The total TBCs and TCs in the sugar-free gummy jellies decreased due to the addition of phycocyanin and UV irradiation treatment, which indicated that phycocyanin and UV irradiation have certain antibacterial effects and can prevent the breeding of micro-organisms.

### 3.4. Changes to Sugar-Free Gummy Jellies in the Accelerated Storage Process

#### 3.4.1. Texture Properties

**Figure 5:**
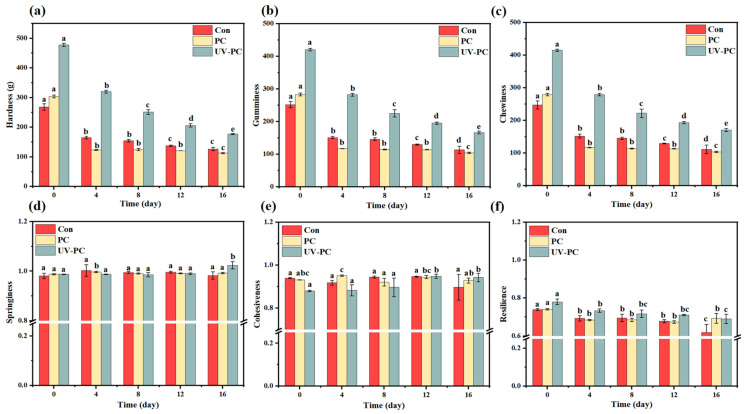
Texture results for *Undaria pinnatifida* sugar-free gummy jellies during accelerated storage: (**a**) hardness; (**b**) gumminess; (**c**) chewiness; (**d**) springiness; (**e**) cohesiveness; (**f**) resilience. Different lowercase letters indicated significant differences (*p* < 0.05).

Texture analysis is a classical method widely used in food texture assessment to provide information on the mechanical properties of samples during deformation [31]. Hardness is the force required to deform the sample to a certain extent, and it is also the internal binding force that characterizes the ability of the sample to retain its shape. Figure 5 shows the changes in the texture parameters of sugar-free gummy jellies under UV irradiation, including their hardness, gumminess, chewiness, springiness, cohesiveness, and resilience. Compared with those of the control group and PC group, the chewiness, gumminess, and hardness of the PC-UV group were considerably enhanced. The hardness was enhanced from 268.4 ± 11.0 g to 477.9 ± 5.2 g. This is explained by the fact that after UV irradiation, the sample produced a large number of free radicals, creating a peroxide environment, which decomposed the phycocyanin polymer and cross-linked with the UP pulp, thus enhancing the gel network structure [32]. Chewiness is the energy required for chewing and is connected with hardness, springiness, and cohesiveness. In this study, chewiness was found to increase significantly from 247.4 ± 12.2 to 415.1 ± 3.1. For similar products, chewiness values have previously been reported to be around 200 [21], in keeping with the data in the present work on gummy jellies. Nearly every structural parameter showed the same general trend. It was confirmed that UV irradiation can significantly change the texture characteristics of sugar-free gummy jellies and provide a better taste for consumers.

Figure 5 also shows the effect of storage time on the texture characteristics of sugar-free gummy jellies. The hardness, gumminess, and chewiness of the sugar-free gummy jellies gradually decreased with an increase in storage time. Kayanna et al. also found that all texture parameters of gummy jelly, including its hardness, springiness, cohesiveness, gumminess, and chewiness, decreased significantly by amounts in the ranges of 2–10% and 5–14% after storage for 7 and 14 days [33]. This phenomenon can be explained by the gradual degradation of the network structure of gelatin and the loose spatial structure during the storage process in a high-temperature and high-humidity environment, which affected the organizational structure of gelatin floss products. Liu et al. indicated that microbial growth during accelerated storage also damaged the internal structure of the gummies, affecting their texture properties [21]. In addition, with an increase in storage time, the springiness, cohesiveness, and resilience of the samples showed little change. The springiness and cohesiveness of the UP sugar-free gummy jellies reached their maximum when the storage time reached the 16th day in the UV-PC group. This was mainly due to the hydrogen bonding between phycocyanin and UP pulp after UV irradiation, which formed a tighter spatial network structure inside the products.

#### 3.4.2. Color

The color differences for the sugar-free gummy jellies at 0, 4, 8, 12, and 16 days of accelerated storage are shown in Table 1. The addition of phycocyanin made the brightness (L*) values of the PC group and the UV-PC group lower than that of the control group. Previous studies also reported lower L* values in candies enriched with *Vitis vinifera* grape capsules [34]. Similarly, the a* and b* values of the sugar-free gummy jellies were significantly changed by the addition of phycocyanin. The significant difference in the a* value and b* value between the PC group and the UV-PC group can be attributed to the change in the structure of phycocyanin in the sugar-free gummy jellies after UV irradiation, resulting in a change in its color.

With the extension of the storage period, the brightness of the control group decreased the fastest, and the brightness of the PC group and UV-PC group decreased slowly. However, the a* value and b* value of the sugar-free gummy jellies showed an increasing trend. This can be explained by the fact that the brightness of the control group without phycocyanin was mainly determined by the color change of the UP pulp; after a long time in storage, the UP pulp turned brown and deepened in color, resulting in a significant decrease in brightness. Similarly, the a* value and b* value changes for the sugar-free gummy jellies were mainly due to UP pulp browning. The main reason for the difference in color between the PC group and the UV-PC group was that the phycocyanin decomposed and the color changed after UV irradiation. A color difference (ΔE) between 0 and 1.5 is generally considered small and nearly imperceptible to the human eye. A ΔE range of 1.5 to 5 indicates a noticeable color difference, while a ΔE value above 5 indicates a clearly visible change in color [35]. After 16 days of storage, the ΔE value of the Con group was 13.22 ± 0.04, the ΔE value of the PC group was 1.95 ± 0.05, and the ΔE value of the UV-PC group showed little change, remaining at 1.29 ± 0.06. This suggested that the color of the UV-PC group remained stable.

#### 3.4.3. Water Migration

**Figure 6:**
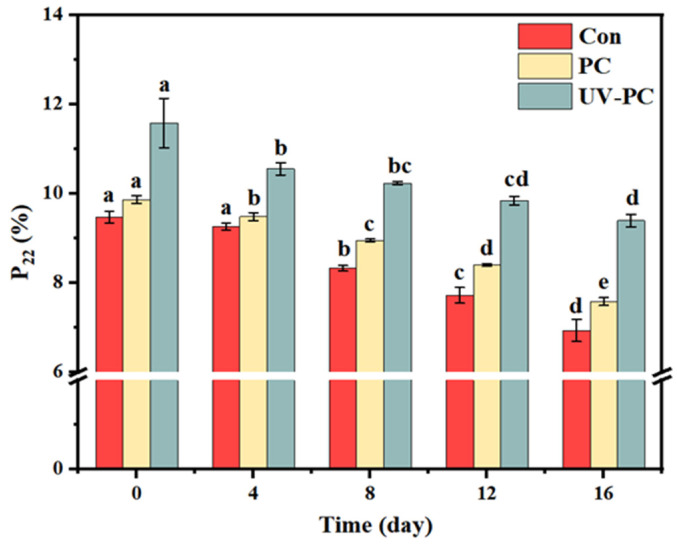
The proportion of immovable water in *Undaria pinnatifida* sugar-free gummy jellies during accelerated storage. Different lowercase letters indicated significant differences (*p* < 0.05).

In a gel network structure, immovable water is considered to be gel-network-bound water, reflecting the structure of the gel network [36]. The moisture change for the sugar-free gummy jellies during accelerated storage is shown in Figure 6. The results showed that the proportion of immovable water decreased, but the proportion of immovable water in the UV-PC group was still higher. This suggests that UV treatment may have a stabilizing effect on the gel network, preventing a significant reduction in water binding capacity during storage. The reduction in the immovable water content may be intricately linked to the evolution of the microstructure of sugar-free gummy jellies. This indicated that the structure of the gel network of the sugar-free gummy jellies gradually loosened as the interactions between the gel-forming components, such as proteins and polysaccharides, weakened with the prolongation of the storage time. This loosening of the network reduced its ability to retain water, leading to a decrease in the proportion of immovable water. However, the UV-PC group, which underwent UV irradiation treatment, exhibited a more stable network, likely due to enhanced cross-linking or other structural modifications caused by the UV irradiation, which helped retain more water within the gel matrix. These findings suggest that as the storage time progressed, the structural integrity of the gel network gradually diminished, weakening its binding capacity for water. The intricate interplay between the substrate and moisture distribution can provide nuanced insights into factors that influence the texture and quality of sugar-free gummy jellies over time, highlighting the crucial influence that compositional modifications, such as UV irradiation, and storage circumstances have on the physicochemical characteristics of sugar-free gummy jellies [26].

#### 3.4.4. Phycocyanin Content

**Figure 7:**
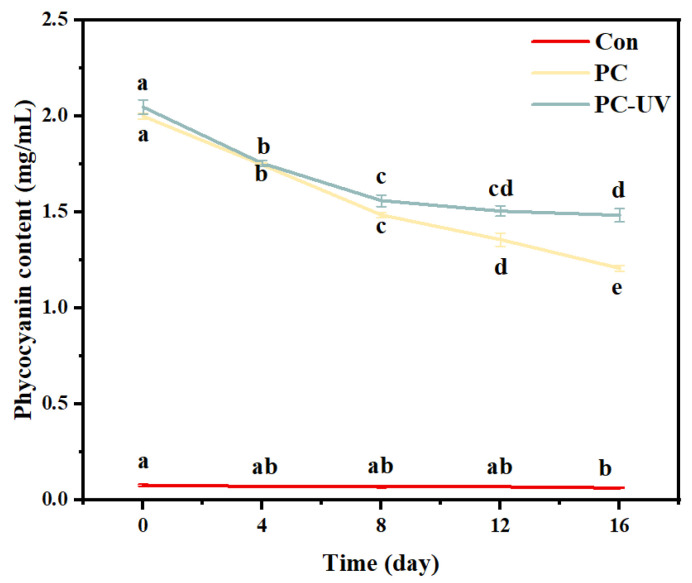
Phycocyanin content of *Undaria pinnatifida* sugar-free jelly during accelerated storage. Different lowercase letters indicated significant differences (*p* < 0.05).

Phycocyanin is easily degraded under high temperature and high humidity during accelerated storage. The phycocyanin content in the sugar-free gummy jellies during the accelerated storage process was determined, and the result is shown in Figure 7. The phycocyanin content decreased with the extension of the accelerated storage process, and there was a significant difference. However, at the end of the accelerated storage process, the phycocyanin content was still retained in both the PC and UV-PC groups, with values of 1.21 ± 0.02 mg/mL and 1.48 ± 0.03 mg/mL, respectively. The corresponding phycocyanin retention rates were 60% and 72%. This demonstrated the effectiveness of the sugar-free gummy jellies in preserving phycocyanin. At the same time, this also showed that the phycocyanin content in the UV-PC group was higher. This was because the phycocyanin in the UV-PC group had already been decomposed at the beginning, and it tended to be stable. However, the phycocyanin in the PC-group decomposed faster under high-temperature and high-humidity conditions. This result can be attributed to the protective effect of UV irradiation treatment. de Moura et al. found that during storage, jelly candy containing microparticles obtained through dripping–extrusion retained 73% of its anthocyanins [37].

## 4. Conclusions

This study demonstrated that the incorporation of phycocyanin under UV irradiation significantly improved the properties of UP pulp-based sugar-free gummy jellies. UV irradiation-assisted phycocyanin enhanced the sensory appeal, increased the hardness from 268.4 ± 11.0 g to 477.9 ± 5.2 g, and boosted the proportion of immovable water from 6.17 ± 0.66% to 9.52 ± 0.77%. Additionally, it improved the microbial stability of the gummy jellies. During the accelerated storage process, UV irradiation-assisted phycocyanin slowed changes in the texture, color, and phycocyanin content, contributing to the jellies’ overall stability. These findings highlight the potential of UP pulp and phycocyanin as key ingredients for developing sugar-free gummy products with improved quality and shelf-life. Future studies should focus on evaluating the nutritional profile of the gummies, investigating how the sugar-free gummy jelly matrix affects phycocyanin bioaccessibility considering its digestive properties, and exploring the application of this method in other functional food products.

## Figures and Tables

**Table 1:** Color changes in sugar-free gummy jellies during accelerated storage.

Sample	Time (Day)	L*	a*	b*	ΔE
Con	0	28.29 ± 0.09 ^Aa^	−1.01 ± 0.02 ^Aa^	2.31 ± 0.09 ^Aa^	-
	4	25.49 ± 0.24 ^Ab^	−0.65 ± 0.03 ^Ab^	2.51 ± 0.09 ^Ab^	2.83 ± 0.15
	8	20.36 ± 0.08 ^Ac^	−0.25 ± 0.03 ^Ac^	2.80 ± 0.06 ^Ac^	7.98 ± 0.03
	12	17.63 ± 0.22 ^Ad^	0.32 ± 0.04 ^Ad^	3.63 ± 0.04 ^Ad^	10.82 ± 0.14
	16	15.36 ± 0.12 ^Ae^	0.24 ± 0.02 ^Ae^	4.74 ± 0.12 ^Ae^	13.22 ± 0.04
PC	0	27.43 ± 0.19 ^Ba^	0.24 ± 0.04 ^Ba^	0.45 ± 0.03 ^Ba^	-
	4	26.56 ± 0.11 ^Bb^	0.35 ± 0.03 ^Bb^	0.62 ± 0.04 ^Bb^	0.89 ± 0.08
	8	26.52 ± 0.22 ^Bb^	0.45 ± 0.04 ^Bc^	0.76 ± 0.02 ^Bc^	0.98 ± 0.03
	12	26.28 ± 0.14 ^Bb^	0.66 ± 0.03 ^Bd^	0.80 ± 0.01 ^Bc^	1.27 ± 0.05
	16	25.67 ± 0.24 ^Bc^	0.67 ± 0.02 ^Bd^	1.17 ± 0.03 ^Bd^	1.95 ± 0.05
UV-PC	0	27.30 ± 0.17 ^Ba^	1.18 ± 0.03 ^Ca^	0.13 ± 0.02 ^Ca^	-
	4	27.44 ± 0.14 ^Ca^	1.35 ± 0.04 ^Cb^	0.24 ± 0.03 ^Cb^	0.25 ± 0.03
	8	26.70 ± 0.30 ^Bb^	1.59 ± 0.02 ^Cc^	0.33 ± 0.03 ^Cc^	0.75 ± 0.13
	12	26.30 ± 0.08 ^Bc^	1.76 ± 0.03 ^Cd^	0.42 ± 0.07 ^Cd^	1.19 ± 0.10
	16	26.24 ± 0.14 ^Cc^	1.80 ± 0.08 ^Cd^	0.52 ± 0.04 ^Ce^	1.29 ± 0.06

Data are expressed as mean ± standard deviation (*n* = 3), with different letters (uppercase or lowercase) indicating significant differences (*p* < 0.05).

## Data Availability

The original contributions presented in this study are included in the article; further inquiries can be directed to the corresponding author.
